# Traumatic Luxation of the Foot

**Published:** 1882-05

**Authors:** 


### Clinical Reports.

---

### ARTICLE V.

### TRAUMATIC LUXATION OF THE FOOT.

N. Wokotawia, æt. fifteen, was admitted on October 15, 1881. He states that while skating, in the winter of 1880, he fell and hurt his right foot. The ankle joint had been much swollen at that time, especially about the external malleolus, and there was a good deal of pain, but no deformity. The doctor called to attend him applied cold water dressing. Three days later, erysipelas having set in, the doctor made some incisions on the outside of the ankle, and thick mattery discharges escaped. About the same time a few abscesses, communicating together, opened spontaneously on the inside of the foot and lower part of the foot and lower part of the gastrocnemius muscle, giving rise to several fistulas. The patient lay sick from four to five months, during which time these fistulas healed, and there had been no caries of the bone. When the patient left his bed, there was no anomaly in the shape of the foot, which was straight, and he was able to walk. The foot became twisted later, however, and subluxation of the knee-joint took place, as subsequent examinations showed.

Actual condition:

Patient well developed for his age. Internal organs normal. Both lower extremities, from the anterior spine of the ilium to the malleoli, were equal in length. The right foot showed a medium degree of plantar flexion, the digital extremity of the right foot being adducted and a little flexed. Active and passive motion were impossible in the ankle joint. The internal malleolus was enlarged, but not protruded. On the inner and on the outer side of the ankle, a scar was left, which adhered to the bone. There was another scar at the point of attachment of the tendon Achillis, from a bedsore caused by decubitus.

Operation:

October 20, patient was anæsthetized, and trials were made at straightening the foot, but failed. Accordingly, on the 26th, they proceeded to cut. They removed a piece of the malleolus with a chain saw, resecting a piece of the bone, and bringing the remaining pieces together, thus straightening the foot. The wound was dressed with iodoform, and covered with an Argantin bandage.

Course:

October 27, temperature normal; dressings, saturated with blood serum, were not changed. The Argantin bandage was reapplied. On the second day of November, the temperature was normal, the discharge had stopped, and the dressings were dry. November 4, bandage changed. Except a few granulations, the rest of the wound had healed. Drainage tube was removed, iodoform and Argantin bandage reapplied. November 21, the temperature had been normal thus far, and there had been no pain. November 22, wound was entirely healed, and united nicely.

